# Migraine days and body mass index (BMI) in a series of Japanese migraineurs

**DOI:** 10.1186/1129-2377-14-S1-P127

**Published:** 2013-02-21

**Authors:** T Takeshima, S Kikui, S Yamashita, Y Tominaga, S Tominaga

**Affiliations:** 1 Neurology, Headache Center, Tominaga Hospital, Japan; 2 Neurosurgery, Tominaga Hospital, Japan

## 

Chronification of migraine headache is one of the most important issues. We analyzed possible association of migraine chronification and obesity in a Japanese series.

## Subjects and methods

We have examined 2662 headache sufferers from February 2010 to March 2012 in our Headache Center. We interviewed all patients with structured questionears. Height and body weight were recorded. Types of headache were determined in accordance with ICHD-II criteria. 1356 subjects had migraine (M:F=349:1007; 152 with aura, 1204 without aura). Mean age of migraineurs was 37.8 ±15.0 (SD) years old. According to BMI, subjects were categorized to five ranks, i.e., underweight (BMI< 18.5), normal (18.5-24.9), overweight (25-29.9), obese (30-34.9), and morbid obese (>35). Average headache days and migraine days of recent three months were recorded. The data were analyzed with chi-square test and one-way ANOVA.

## Results

94 out of 231 underweight migraineurs (40.1%), 383 of 945 normal-weight ones (40.6%), 61 of 139 overweight ones (43.9%), 15 of 31 obese ones (48.4%), and 9 of 10 morbid obese ones (90.0%) had more than 15 headache days and 8 migraine days (p<0.05, Pearson's chi-square test). Mean headache days were 13.9 ± 0.7 (SE), 13.7 ± 0.35, 14.8 ± 0.9, 15.2 ± 2.0 and 20.5 ± 2.5 days/month in underweight, normal, overweight, obese and morbid obese migraineurs, respectively (N.S., ANOVA). Mean migraine days were 6.4 ± 0.4, 6.5 ± 0.2, 8.2 ± 0.5, 9.5 ± 1.3 and 12.5 ±1.3, respectively (p< 0.0001, ANOVA). BMI was significant risk factor after age adjustment (p<0.001, partial correlation analysis).

## Conclusion

Overweight or obese migraineurs tended to have more migraine days than normal or underweight migraineurs in a Japanese series. Although the number of obese migraineurs is not large, obesity is a possible correctable risk factor for migraine chronification in Japanese population.

